# Extended Superspheres for Shape Approximation of Near Polyhedral Nanoparticles and a Measure of the Degree of Polyhedrality

**DOI:** 10.3390/nano6020027

**Published:** 2016-02-02

**Authors:** Susumu Onaka

**Affiliations:** Department of Materials Science and Engineering, Tokyo Institute of Technology, 4259 Nagatsuta, Yokohama 226-8502, Japan; onaka.s.aa@m.titech.ac.jp

**Keywords:** particle, precipitate, elastic-strain energy, equilibrium shape, polyhedron, supersphere

## Abstract

Crystalline nanoparticles or nanoprecipitates with a cubic structure often have near polyhedral shapes composed of low-index planes with {100}, {111} and {110}. To consider such near polyhedral shapes, algebraic formulas of extended superspheres that can express intermediate shapes between spheres and various polyhedra have been presented. Four extended superspheres, (i) {100} regular-hexahedral; (ii) {111} regular-octahedral (iii) {110} rhombic-dodecahedral and (iv) {100}-{111}-{110} rhombicuboctahedral superspheres are treated in this study. A measure ∏ to indicate the degree of polyhedrality is presented to discuss shape transitions of the extended superspheres. As an application of ∏ superspherical coherent precipitate is shown.

## 1. Introduction

**Figure 1:**
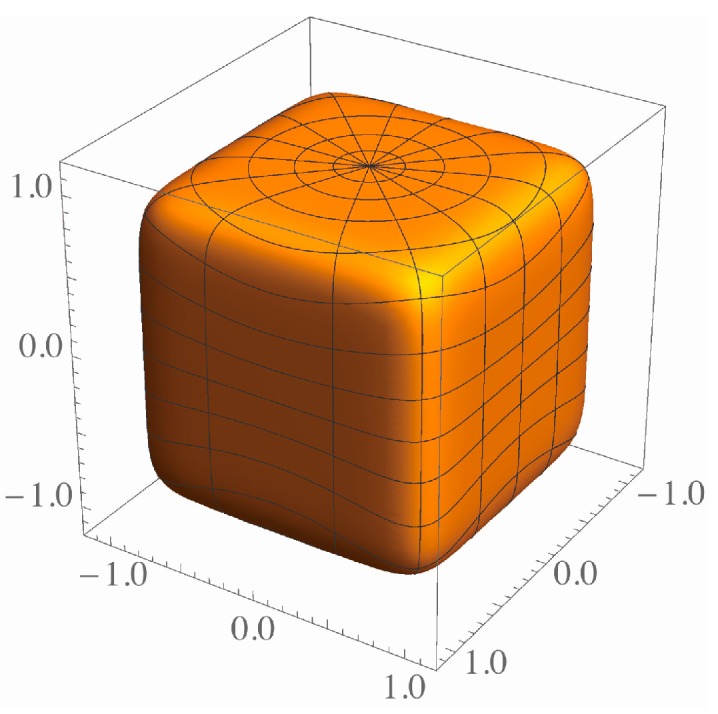
The shape given by Equation (1) for p=8 and R=1.

To consider intermediate shapes of nanoparticles or nanoprecipitates between a sphere and cube, a solid figure called a supersphere was discussed in previous studies [1,2]. An equation describing the supersphere is

(1) $${\left|x/R\right|}^{p}+{\left|y/R\right|}^{p}+{\left|z/R\right|}^{p}=1\;(R>0,p\geq 2)$$

and expresses a sphere with radius *R* when *p* = 2 and a cube with edges 2*R* as p→∞. It is reported in [3] that Equation (1) first appeared in a paper by Gabriel Lamé, the 19th century French mathematician. When |x|>|y|,|z| and p→∞, Equation (1) becomes |x/R|=1. This explains the reason why the limit for Equation (1) gives a cube surrounded by three sets of parallel planes |x|=R, |y|=R and |z|=R [4]. Shape transition from a sphere to cube can be represented by increasing *p*. Figure 1 shows the shape given by Equation (1) for p=8 and R=1.

In the case of crystals having cubic structures, nanoparticles or nanoprecipitates often have near polyhedral shapes composed of low-index planes with {100}, {111} and {110} [5,6]. These planes have lower values of the surface-energy density and the anisotropy of surface-energy density affects the equilibrium shapes [7,8]. The anisotropy of elastic moduli also affects equilibrium shapes of misfit nanoprecipitates in alloys [9,10]. The nearly polyhedral shapes of the nanoparticles or nanoprecipitates have been often explained by such anisotropies [7,8,9,10].

Onaka recently extended Equation (1) and derived algebraic formulas to describe intermediate shapes between spheres and various convex polyhedra [4,11]. Now superspheres mean shapes intermediate between spheres and various polyhedra [4]. The original supersphere given by Equation (1) can be called a cubic or hexahedral supersphere to distinguish these from other superspheres such as an octahedral supersphre. The extended superspheres have been used to approximate various near polyhedral shapes of nanoparticles and nanoprecipitates [8,12].

The superspherical-shape approximation is useful to discuss various near polyhedral shapes of crystalline nanomaterials [1,8,10,12,13,14,15]. The extended superspheres are also treated in mechanics as possible shapes of inclusions and pores in materials [16,17,18,19,20,21]. When we use the superspherical-shape approximation, a measure of the degree of polyhedrality is needed to discuss the shape transitions [2,8,10]. Equations to describe the extended superspheres essentially have the same form as Equation (1) [4,11]. Since the shapes of the extended superspheres also change from spheres to polyhedra with increasing the power exponent *p* in Equation (1), *p* has been hence used as the measure of the degree of polyhedrality[2,4]. Instead of *p* without the upper bound, a parameter η given by

(2) $$\eta =\sqrt{2}\cdot 2^{\left(-1/p\right)}$$

which satisfies η=1 when p=2 and $\eta =\sqrt{2}$ when p→∞ has also been used [2]. Since η is the ratio of the maximum to minimum radii on the cross-sections of the cubic supersphere, it is a convenient parameter to grasp the shape transition [2]. However, for the other extended superspheres, η does not generally have such geometrical meaning. As will be shown later, *p* or η is not appropriate as a common measure of the degree of polyhedrality of the extended superspheres. In the present paper, we propose a new parameter Π as the measure of the degree of polyhedrality. As an application of Π, we will show the precipitate-shape dependence of elastic-strain energy of a material containing a superspherical coherent precipitate.

## 2. Equations of Extended Superpheres

Here we show equations of the extended superspheres that become polyhedra composed of {100}, {111} and {110} as the limiting shapes [4].

• {100} Regular-hexahedral supersphere:

(3a) $${\left[h_{\text{hexa}}\left(x,y,z\right)\right]}^{1/p}=R$$

where

(3b) $$h_{\text{hexa}}\left(x,y,z\right)={\left|x\right|}^{p}+{\left|y\right|}^{p}+{\left|z\right|}^{p}$$

Here the *x*, *y* and *z* axes mean 〈100〉 of a crystal with a cubic structure. Equations (3) are the same as Equation (1) for the original supersphere.

• {111} Regular-octahedral supersphere:

(4a) $${\left[h_{\text{octa}}\left(x,y,z\right)\right]}^{1/p}=R$$

where

(4b) $$h_{\text{octa}}\left(x,y,z\right)={\left|x+y+z\right|}^{p}+{\left|-x+y+z\right|}^{p}+{\left|x-y+z\right|}^{p}+{\left|x+y-z\right|}^{p}$$

• {110} Rhombic-dodecahedral supersphere:

(5a) $${\left[h_{\text{dodeca}}\left(x,y,z\right)\right]}^{1/p}=R$$

where

(5b) $$h_{\text{dodeca}}\left(x,y,z\right)={\left|x+y\right|}^{p}+{\left|x-y\right|}^{p}+{\left|y+z\right|}^{p}+{\left|y-z\right|}^{p}+{\left|x+z\right|}^{p}+{\left|x-z\right|}^{p}$$

• {100}-{111}-{110} Polyhedral supersphere:

(6) $${\left[h_{\text{hexa}}\left(x,y,z\right)+\frac{1}{a^{p}}h_{\text{octa}}\left(x,y,z\right)+\frac{1}{b^{p}}h_{\text{dodeca}}\left(x,y,z\right)\right]}^{1/p}=R$$

**Figure 2:**
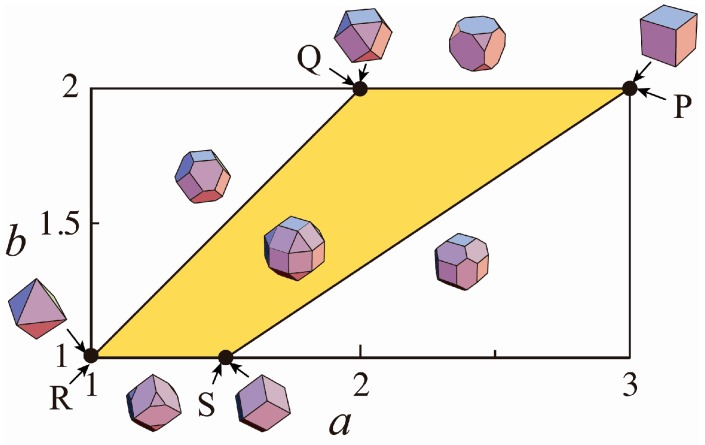
Diagram showing the variations of polyhedra composed of {100}, {111} and {110} as the limiting shapes of the extended superspheres The parameters *a* and *b* are those for determining the ratios of the {100}, {111} and {110}. The points P, R and S respectively correspond to the {100} hexahedron, the {111} octahedron and the {110} dodecahedron. Polyhedra composed of one or two of the crystallographic planes can be shown around the quadrilateral surrounded as shown in the insets. The {100}-{111}-{110} polyhedra with different ratios of the three crystallographic planes are expressed inside of the quadrilateral.

This Equation (6) is an equation combining $h_{\text{hexa}}$, $h_{\text{octa}}$ and $h_{\text{dodeca}}$ and gives superspheres which become the {100}-{111}-{110} polyhedra as the limiting shapes. When p→∞, we find that the innermost surfaces of the polyhedra are retained to form the combined polyhedron among the three polyhedra given by ${\left[h_{\text{hexa}}\left(x,y,z\right)\right]}^{1/p}=R$, ${\left[h_{\text{octa}}\left(x,y,z\right)\right]}^{1/p}=aR$ and ${\left[h_{\text{dodeca}}\left(x,y,z\right)\right]}^{1/p}=bR$ [4]. The parameters *a* and *b* are those for determining the ratios of the {100}, {111} and {110} surfaces of the limiting shapes. Figure 2 shows the shapes of the polyhedra as a function of *a* and *b* [4]. In the present study, we will consider the supersphere with $a=\left(2\sqrt{2}-1\right)$ and $b=\sqrt{2}$ that becomes a rhombicuboctahedron (RCO) with six square {100}, eight equilateral-triangular {111} and twelve square {110} when p→∞.

## 3. Geometrical Changes in Superpheres and Measure of the Degree of Polyhedrality

Sphere is the solid figure having the minimum surface area under constant volume. The shape transitions of the extended superspheres from spheres to polyhedra cause the increase of surface area when the volume is kept constant. When A and V respectively denotes the surface area and volume of a solid figure, N given by

(7) $$N=A/V^{2/3}$$

is a measure of the surface area under constant volume [8,12]. The cube of N,

(8) $$S=N^{3}=A^{3}/V^{2}$$

is known as the Steinitz number that has been used to discuss geometrical characteristics of polyhedral [22,23].

**Figure 3:**
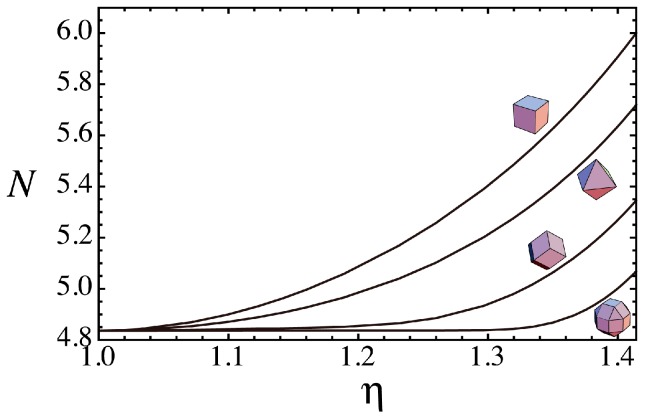
The relationship between $N=A/V^{2/3}$ and η for the hexahedral, octahedral and dodecahedral superspheres respectively given by Equations (3)–(5) and rhombicuboctahedral (RCO) supersphere given by Equation (6) with $a=\left(2\sqrt{2}-1\right)$ and $b=\sqrt{2}$. The results for the superspheres are shown with insets of the polyhedral shapes when $\eta =\sqrt{2}$.

The shape transitions of the extended superspheres from spheres to various polyhedra represented by the relationship between N and η are shown in Figure 3 for the hexahedral, octhedral and dodecahedral superspheres respectively given by Equations (3)–(5) and RCO supersphere given by Equation (6) with $a=\left(2\sqrt{2}-1\right)$ and $b=\sqrt{2}$. In Figure 3, the results are shown with the insets of the polyhedral shapes when $\eta =\sqrt{2}$. Although N monotonically increases from that for a sphere $N\left(\text{sphere}\right)={\left(36\pi \right)}^{1/3}\approx 4.84$ to those of the polyhedra, the change in *N* with increasing η is not the same among the four superspheres.

To compare the changes in N from spheres to the polyhedra, we introduce the normalized change Π given by:

(9) $$\Pi \left(\eta \right)=\frac{N\left(\eta \right)-N\left(\text{sphere}\right)}{N\left(\text{polyhedron}\right)-N\left(\text{sphere}\right)}$$

**Figure 4:**
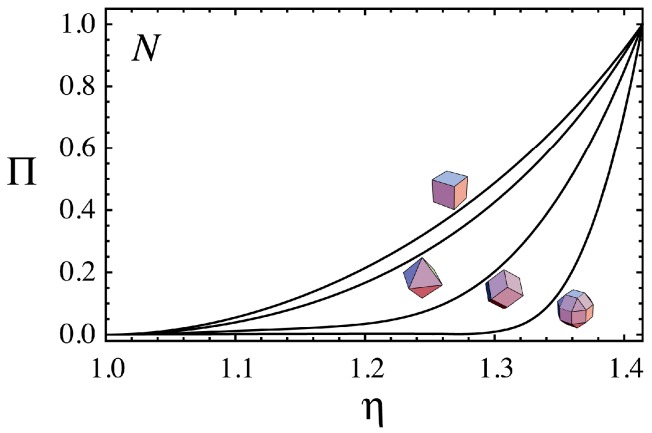
The relationship between Π given by Equation (9) and η for the hexahedral, octahedral and dodecahedral and RCO superspheres, where Π is a function of N.

For a sphere and polyhedron, we have Π=0 and Π=1 respectively. Figure 4 shows the changes in Π as a function of η for the hexahedral, octhedral, dodecahedral and RCO superspheres. The η dependence of Π shown in Figure 4 is quite different among the superspheres. The shape transitions from spheres to the polyhedra are delayed in the order of the hexahedral, octhedral, dodecahedral and ROC superspheres. For example, although the hexahedral supersphere with η≈1.3(p≈8) has a near polyhedral shape as shown in Figure 1, the RCO supersphere with the same value of η still has the values of N and Π almost the same as those of a sphere. The shape transition of the RCO supersphere is not noticeable at η≈1.3.

**Figure 5:**
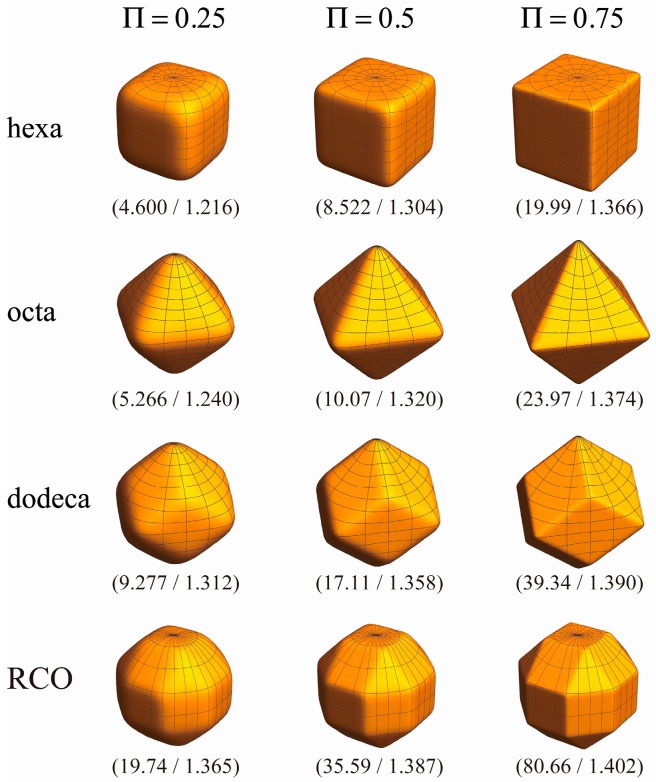
The shapes of the hexahedral, octahedral, dodecahedral and RCO superspheres at various values of Π. The values in a parenthesis separated by a slash are those of p (**left**) and η (**right**) for each shape.

The parameter Π given by N is a more reasonable measure of the degree of polyhedrality than p or η. Figure 5 shows the shape variations of the superspheres at various values of Π. The values in a parenthesis separated by a slash in Figure 5 are those of p (left) and η (right) for the shape. For the initial shape transitions at lower Π, p and η at the same Π are much different among the various superspheres. For example, when Π=0.25, we have 1.216 and 1.365 for the values of η for the hexahedral and RCO superspheres, respectively.

**Figure 6:**
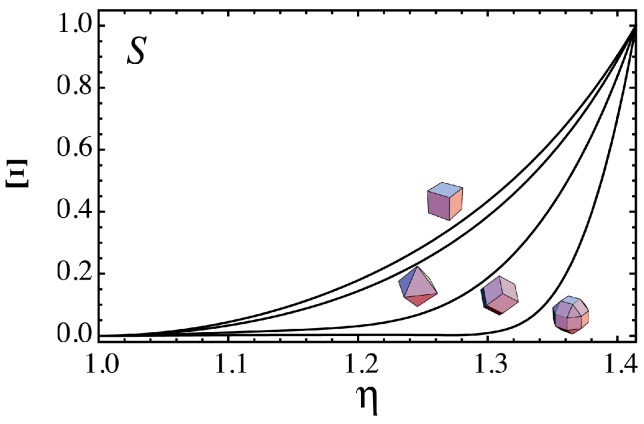
The relationship between Ξ and η for the hexahedral, octahedral and dodecahedral and RCO superspheres, where Ξ is a function of the Steinitz number S. It is interesting to note that this Ξ−η relation is very similar to the Π−η relation (Figure 4).

We have considered Π given by N, since N has a clear geometrical-meaning, the surface area under constant volume. If we use the Steinitz number S given by Equation (8) instead of N, the normalized change Ξ given by

(10) $$\Xi \left(\eta \right)=\frac{S\left(\eta \right)-S\left(\text{sphere}\right)}{S\left(\text{polyhedron}\right)-S\left(\text{sphere}\right)}$$

is obtained. It is interesting to note that even if we consider Ξ given by S, we have the Ξ−η relation (Figure 6) which is very similar to the Π−η relation (Figure 4).

## 4. Example of Applications of the Polyhedrality

It is well known that the total surface or interface energy changes when shapes of particles or precipitates change. Similarly, mechanical energy such as elastic strain energy is also changed by shape changes of misfit precipitates in alloys [9,10,14]. As an example of applications of the polyhedrality Π, here we show the precipitate-shape dependence of the elastic-strain energy for coherent precipitates in a matrix.

**Figure 7:**
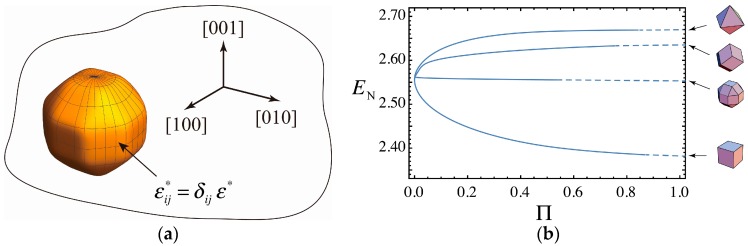
(**a**) Schematic illustration showing a superspherical coherent precipitate in a matrix with a cubic structure. The precipitate has a purely dilatational misfit strains $\varepsilon_{ij}^{*}=\delta_{ij}\varepsilon^{*}$ and causes elastic strains in the material containing the precipitate. (**b**) The precipitate-shape dependence of the elastic-strain energy shown by the relationship between the normalized elastic-strain energy $E_{N}$ and the polyhedrality Π. The results for the precipitate shapes of the hexahedral, octhedral, dodecahedral and RCO superspheres are shown.

The situation to consider the elastic-strain energy is summarized in Figure 7a. The matrix with a face-centered cubic structure contains a coherent precipitate having the same elastic moduli as the matrix. Those of the extended superspheres treated in the present study give possible shapes of the precipitate. The precipitate has a purely dilatational misfit strains $\varepsilon_{ij}^{*}=\delta_{ij}\varepsilon^{*}$ and causes elastic strains in the material including the precipitate [24], where $\delta_{ij}$ the Kronecker delta giving the components of the strain tensor. The elastic strain energy E due to the superspherical precipitate can be numerically calculated as a function of the misfit strain $\varepsilon^{*}$, the volume of the precipitate V, the elastic modulus of the material including their anisotropy and the shape factor of the superspherical precipitate such as p, η or Π [10]. Figure 7b shows the relationship between the normalized elastic-strain energy $E_{N}$ and the polyhedrality Π, where

(11) $$E_{N}=E/\left[C_{44}{\left(\varepsilon^{*}\right)}^{2}V\right]$$

and $C_{44}$ is one of the elastic modulus of the cubic material. The anisotropy of elastic moduli of Cu is used to show Figure 7b. $E_{N}$ for precipitates with sharp edges are evaluated by extrapolation [10] as shown by broken lines in Figure 7b.

As shown in Figure 7b, the values of $E_{N}$ for the {100}-{111}-{110} RCO supersphere are always almost the same as that for a sphere at Π=0. The {100} hexahedral and the {111} octahedral polyhedra have the minimum and the maximun $E_{N}$ among the polyhdron. Including the {110} dodecahedral superspheres, the increasing and decreasing behavior of $E_{N}$ for these superspheres with shape transitions from the sphere are similar when we adopt Π as a measure of the degree of polyhedrality. The introduction of the polyhedrality Π is convenient to discuss such changes in $E_{N}$ with the shape transitions. Other applications of Π will be shown in our future work.

## 5. Conclusions

Crystalline nanoparticles or nanoprecipitates often have near polyhedral shapes composed of low-index planes. Intermediate shapes between spheres and various convex polyhedra can be approximated with the concept of the extended superspheres. Four extended superspheres, (i) {100} regular-hexahedral; (ii) {111} regular-octahedral, (iii) {110} rhombic-dodecahedral and (iv) {100}-{111}-{110} rhombicuboctahedral superspheres have been treated in this study. A measure Π to indicate the degree of polyhedrality has been presented to discuss shape transitions of the extended superspheres. As an application of Π, the precipitate-shape dependence of elastic-strain energy of a material containing the superspherical coherent precipitate has been shown.
